# The cardioprotective and anti-inflammatory effect of inhaled nitric oxide during Fontan surgery in patients with single ventricle congenital heart defects: a prospective randomized study

**DOI:** 10.1186/s40560-022-00639-y

**Published:** 2022-10-13

**Authors:** Jacek Kolcz, Elzbieta Karnas, Zbigniew Madeja, Ewa K. Zuba-Surma

**Affiliations:** 1 Department of Pediatric Cardiac Surgery, Jagiellonian University Medical College, Wielicka 265 Street, 30-663 Krakow, Poland; 2 Department of Cell Biology, Faculty of Biochemistry, Biophysics and Biotechnology, Jagiellonian University, Krakow, Poland

**Keywords:** Inflammatory mediators, Proteases, Congenital heart defect, Cardiopulmonary, Nitric oxide, Fontan surgery

## Abstract

**Background:**

Fontan surgery with cardiopulmonary bypass (CPB) causes tremendous systemic stress and inflammatory responses, affecting postoperative organ function, morbidity, and mortality. Although this reaction triggers partially protective anti-inflammatory responses, it is harmful in patients with single ventricle congenital heart defects. Despite decades of research, an effective anti-inflammatory and stress defense strategy is lacking. This study investigated the influence of inhaled nitric oxide (NO) during CPB on early clinical results, including the duration of postoperative respiratory support as a primary outcome and a panel of laboratory analytes.

**Methods:**

In this study, 115 patients were randomized to the Fontan-NO group (*n* = 48) and the Fontan group (*n* = 49). Eighteen patients were excluded from the study. The Fontan-NO group received NO inhaled directly into the oxygenator during CPB. Clinical data were collected, and blood samples were drawn for analysis at repeated intervals. Multiplex assays were used to analyze a proteome profile of molecules involved in stress response, inflammation, metabolic reactions, as well as heart and lung protection.

**Results:**

Fontan-NO patients had significantly shorter respiratory support time with a median of 9.3 h (7.0; 13,2) vs 13.9 h (3.7; 18.5) by the absolute difference of 4.6 h [95% confidence interval, − 30.9 to 12.3; (*p* = 0.03)]. In addition, they have a shorter time in intensive care (*p* = 0.04) and lower pulmonary artery pressure after CPB discontinuation (*p* = 0.04), 4 h (*p* = 0.03) and 8 h (*p* = 0.03) after surgery. Fontan-NO patients also had a lower concentration of lactates (*p* = 0.04) and glucose after separation from CPB (*p* = 0.02) and lower catecholamine index (*p* = 0.042). Plasma factors analysis has shown a significantly higher concentration of interleukin-10, and a lower concentration of interleukin-6, interleukin-8, interleukin-1β, pentraxin, matrix metalloproteinase-8, troponin-I, creatine kinase myocardial band (CK-MB), and insulin in Fontan-NO group.

**Conclusions:**

NO inhaled into the oxygenator during CPB can improve short-term clinical outcomes. It shortens intubation time and intensive care time. It reduces inflammatory response, improves myocardial and lung protection, and diminishes metabolic stress in patients with a single ventricle undergoing Fontan surgery.

*Trial registration number:* The trial was preregistered, supervised, and supported by The Polish National Science Center (NCN/01/B/NZ5/04246).

**Supplementary Information:**

The online version contains supplementary material available at 10.1186/s40560-022-00639-y.

## Background

Single ventricle heart defects are a challenging group of complex congenital heart diseases that require staged surgical treatment, ending with a Fontan procedure. Heart surgery with cardiopulmonary bypass (CPB), hypothermia, and cardioplegic heart arrest causes systemic stress responses leading to endothelial dysfunction, neuroendocrine dysregulation, and deterioration of organs [1, 2]. These are particularly harmful in the increasing population of patients with Fontan circulation [3, 4]). Furthermore, preoperative cyanosis and heart failure with postoperative Fontan physiology predispose patients to an exacerbated systemic inflammatory response, capillary leak syndrome, heart and lung dysfunction, abdominal organ deterioration, and worsening clinical outcomes [5]. At the same time, these factors contribute to extended ventilatory and inotropic support, increased costs and decreased cost-effectiveness of therapy [6, 7]. Therefore, a search for adequate anti-inflammatory and organ protection strategies is essential, particularly in the population undergoing complex procedures with CPB.

Nitric oxide (NO) is a keynote regulator of blood flow and tissue oxygenation. It also controls crucial cardiovascular, respiratory, nervous, and immunological cellular processes. Intrinsic NO deficiency leads to organ and system dysfunction, suggesting that extrinsic NO may have therapeutic applications [8, 9]. During CBP, hemodilution, non-physiological shear stress, and hemolysis related to mechanical damage of erythrocytes increase free hemoglobin concentration in peripheral blood [10, 11]. As a potent NO scavenger, free hemoglobin causes a rapid drop in NO bioavailability in the vascular system, leading to impaired tissue perfusion and organ dysfunction, systemic and local pro-inflammatory activation, ischemia–reperfusion damage and metabolic dysregulation [12, 13]. Previous studies in children with tetralogy of Fallot showed that NO reduced inflammation markers and frequency of low cardiac output in the early postoperative period [14].

Prolonged ventilation support after cardiac surgery is linked with postoperative heart failure and complications that influence intensive care unit (ICU) length of stay and hospitalization time [15, 16]. Furthermore, previous studies revealed that extubation time was related to CPB duration and strongly predicted postoperative morbidity and mortality [17–19]. In the present study, we hypothesized that respiration support time could be influenced by intraoperative NO inhalation into the oxygenator during a critical drop of endogenous NO bioavailability in patients with endothelial dysfunction at baseline. Additionally, we determined the influence of NO on crucial early clinical outcomes and a proteome profile of molecules involved in stress response, inflammation, metabolic reactions, and heart and lung protection.

## Methods

### Study design and Fontan surgery protocol

The study was approved by the Jagiellonian University Ethics Committee (KBET/176/B/2019) and registered with the Polish National Science Committee (NCN 01/B/NZ5/04246). Informed consent was obtained from the patient's parents before enrollment. The sample size determination was based on our preliminary study focused on clinical and laboratory outcomes in Fontan patients. The study revealed a reduction of ventilation time (using synchronized intermittent mandatory ventilation; SIMV) after Fontan surgery with NO inhalation by 4 h on average with a standard deviation of 9.7, a power of 0.80 and *p* < 0.05 [20]. The sample size of 96 subjects (48 in each arm) is sufficient to detect a clinically important difference of 4 h between groups in shortening ventilatory support time (using SIMV), assuming a standard deviation of 9.7, using a two-tailed t-test of difference between means with 80% power and a 5% level of significance.

### Surgical methods

All patients were treated with identical equipment and CPB strategy, including hypothermia, cardioplegic heart arrest [21], and intraoperative steroid (methylprednisolone; 20 mg/kg) administration to the priming solution. None of the patients had ultrafiltration performed. The general anesthesia protocol was identical for all patients. During induction of anesthesia, patients received midazolam (0.15–0.3 mg/kg), fentanyl (5–10 µg/kg) and rocuronium (0.6–1.2 mg/kg). Anesthesia was maintained with midazolam, sufentanil, and neuromuscular blocker. All patients underwent routine Fontan surgery with 3.5 mm fenestration.

### Allocation of patients

After sample size determination, 97 patients were allocated to the Fontan group (did not receive nitric oxide inhalation) or the Fontan-NO group (received NO inhalation) by computer-based block randomization (MS Excel). For this, ten patients in each block were randomized on a 1:1 basis into two groups. Codes were computer generated and sealed in the envelopes. The codes were not accessible to scientific assistants and treatment staff to ensure proper concealment. For each patient randomized, the following available code was used. The scientific assistant passed the sealed envelope on to the perfusionist on the day of surgery, just before the onset of CPB. The gas delivery device and the NO monitor were identically set up in all patients, regardless of the allotted group. The operating theatre and ICU staff were blinded to the study protocol. The perfusionist who obtained the patient's code from the scientific assistant controlled the position of the NO outlet valve. A self-regulating servomechanism maintained NO delivery at 20 ppm in the gas inlet mixture throughout CPB in Fontan-NO patients.

### Blood sample collection and time-points

The parents of 42 patients (18 patients from the Fontan–NO group and 24 from the Fontan group) consented to collect additional blood samples for hematological and proteome analysis.As part of routine intraoperative and postoperative monitoring, peripheral blood (PB) samples were collected before surgery (1), intraoperatively: onset of CPB during cooling (2), after the opening of the cross-clamp (3), during warming after complete reperfusion (4), after discontinuation of CPB (5), and at ICU 4 h (6), 8 h (7), 12 h (8), and 24 h (9) after surgery. Sample collectors, clinical investigators, assistants, and laboratory personnel were unaware of the study protocol. Blood samples were used to determine the concentrations of investigated analytes and hematologic parameters.

### Statistical analysis

The normal distribution of the quantitative data was tested with the Shapiro–Wilk test. Normally distributed quantitative variables were presented as mean and standard deviation (SD). Data not normally distributed were presented using the median, first, and third quartiles (Q1; Q3). Differences between normally distributed data were assessed using a t-test. Data that were not normally distributed were compared with the Mann–Whitney test (including primary endpoint, i.e. SIMV time). Fisher's exact test was used to determine the differences between the groups of categorical data. Repeated measure analysis of variance was used to screen for time effect and interactive effect between time and group. A Bonferroni post hoc analysis was performed to determine a significance level between groups at a particular time-points. Statistically significant variables were selected for multiple regression after excluding collinearity. The stepwise procedure was applied to find predictors of respiratory time in the ICU, hospitalization time, catecholamine index, and duration of effusions. The value of α < 0.05 was considered statistically significant. Statistical analysis was performed using the data analysis software Dell Statistica (version 13; software.dell.com).

### Semiquantitative analyte screening

The Human Cytokine Array Kit and Angiogenesis Array Kit (R&D Systems) evaluated 36 cytokines, chemokines, acute phase proteins, and 55 angiogenesis-related proteins. The experiments were carried out according to the manufacturer's protocol. For screening, plasma samples were mixed with detection antibodies and incubated with arrays containing duplicate spots of capture-labelled antibodies. After washing, the arrays were incubated with streptavidin-conjugated horseradish peroxidase. The chemiluminescent substrate was added, and signals were detected by a MicroChemi analyzer (DNR Bioimaging System). Densitometric studies of the averaged pixel density of duplicate spots were carried out using Quantity One software (Bio-Rad). Relative cytokine levels were calculated compared to control time point 1. An average background signal from negative controls was subtracted from each spot during the analysis. Positive control spots were also included on each membrane to ensure the repetitiveness of each assay.

### Quantitative multiplex measurement of plasma cytokines

Quantitative analysis of proteins expression was performed with Milliplex Map Kit assays (Merck Millipore) based on magnetic detection coupled with the Luminex xMAP platform. A detailed list of Milliplex kits is indicated in Additional file 1: Table S1. Plasma samples were treated according to the manufacturer's protocol. Briefly, 96-well plates with appropriate fluorescent beads, conjugated with antibodies that captured analytes, were incubated with plasma samples. Subsequently, fluorescent-detecting antibodies were added, and the fluorescence level in each well was read using the Bio-Plex 200 system (Bio-Rad) and analyzed using Bio-Plex Manager software (Bio-Rad). Analyte concentration was calculated based on standard curves for protein standards.

## Results

### Clinical results

**Fig. 1:**
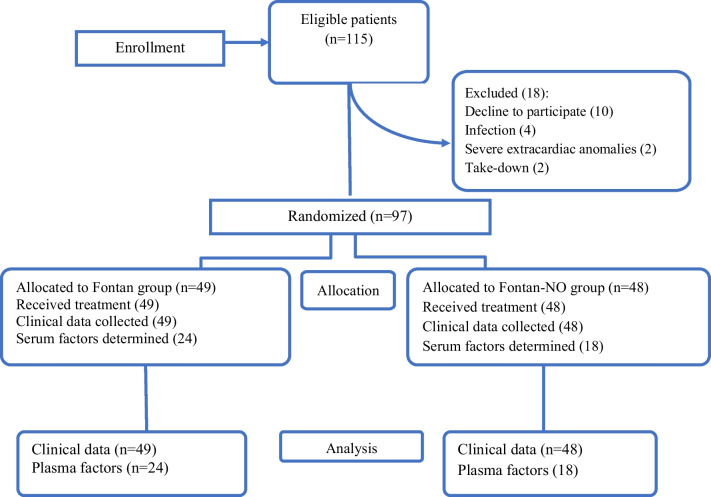
Flow chart of the trial enrollment

The study enrolled 115 patients who underwent elective Fontan surgery for single ventricle defects after initial palliative procedures. Eighteen patients were excluded from the study due to participation refusal, infections, severe extracardiac abnormalities, or procedure changes (Fig. 1). The study participants, aged 1.8 to 3.3 years, consisted of 55 patients with hypoplastic left heart syndrome variants, 29 patients with tricuspid atresia, eight patients with hypoplasia of the right ventricle complex, seven patients with double inlet left ventricle, six patients with double outlet right ventricle with left ventricular hypoplasia, five patients with pulmonary atresia and intact ventricular septum, and five patients with heterotaxy syndrome.

Data on demographic variables and perioperative parameters are presented in Table 1. No differences in age, sex, single ventricle anatomy, cardiopulmonary bypass time, cross-clamp time, heart rate, systolic and diastolic blood pressure, and hospitalization time were observed. Patients in the Fontan-NO group had significantly shorter respiratory support time with a median of 9.3 h (7.0; 13,2) vs 13.9 h (3.7; 18.5) by the absolute difference of 4.6 h [95% confidence interval, -30.9 to 12.3; (*p* = 0.03)]. In addition, they have a shorter time in intensive care (*p* = 0.04) and significantly lower central venous pressure (which in Fontan patients reflects pulmonary artery pressure) after discontinuation of CPB [16.2 (14.1; 21.6) vs 14.2 (9.4; 16.0); *p* = 0.04], four hours after surgery [14.3 (10.6; 19.2) vs 10.9 (9.7; 14.3); *p* = 0.03], and 8 h after surgery [17.11 (11.3; 19.1) vs 11.3 (8.7; 13.1); *p* = 0.03] compared to the Fontan group. Furthermore, they had a lower median post bypass lactate level [3.75 (3.2; 6.5) vs 5.12 (2.9; 7.9); *p* = 0.04], post by-pass glucose level [5.16 (4.1; 5.8) vs 9.3 (7.8; 11.5); *p* = 0.02], lower maximum catecholamine index [3.4 (2.8; 4.1) vs 5.6 (4.6; 6.8); *p* = 0.042], and propensity for shorter duration of effusions [12.5 (10.5; 17.6) vs 15.2 (14.2; 21.4); *p* = 0.05] compared to Fontan patients (Table 1). Administration of NO during CBP significantly improved short-term clinical measures.

**Table 1:** Summary of clinical characteristics of patients enrolled in the study

Variable	Group of patients		*p*
	Fontan (*n* = 49)	Fontan NO (*n* = 48)	
Male; *n* (%)	21 (42.8)	22 (45.8)	0.97
Female; *n* (%)	28 (57.1)	26 (54.2)	
Age in days; mean (SD)	964.6 (228.7)	897.4 (242.4)	0.33
Dominant ventricle; right *n* (%)	22 (44.9)	20 (41.6)	0.90
Preoperative oxygen saturation (%SO_2_); mean (SD)	82.4 (12.3)	81.6 (18.4)	0.36
CPB time (min); mean (SD)	98.5 (13.1)	95.6 (18.3)	0.45
Cross-clamp time (min); mean (SD)	33.7 (6.7)	36.5 (8.3)	0.12
Post-CPB Lactate level (mmol/l); median (Q1; Q3)			
(5)	5.12 (2.9; 7.9)	3.75 (3.2; 6.5)	0.04
(6)	2.87 (1.5; 8.2)	3.21 (1.9; 4.0)	0.69
(7)	5.45 (2.9; 6.7)	4.34 (3.6; 5.1)	0.15
(8)	3.2 (2.5)	2.6 (3.9)	0.44
(9)	1.4 (0.8; 2.1)	1.3 (0.4; 0.6)	0.33
Post-CPB glucose level (mmol/l); median (Q1; Q3)			
(5)	9.3 (7.8; 11.5)	5.16 (4.1; 5.8)	0.02
(6)	7.6 (5.2; 12.4)	7.2 (5.9; 8.1)	0.06
(7)	8.3 (4.4; 12.1)	6.3 (5.1; 8.3)	0.12
(8)	6.8 (3.6; 9.1)	8.1 (7.3; 9.1)	0.62
(9)	6.3 (2.6; 7.2)	6.1 (5.2; 7.3)	0.43
HR (beat/min); median (Q1; Q3)			
(5)	97.4 (93.2; 110.2)	103.9 (94.9; 114.9)	0.25
(6)	113.8 (89.5; 130.4)	94.3 (78,3; 105.9)	0.12
(7)	105.4 (97.1; 131.2)	96.9 (87.2; 113.1)	0.22
(8)	110.5 (92.5; 126.4)	91.6 (82.5; 112.7)	0.08
(9)	108.2 (95.9; 124.9)	106.5 (102.1; 109.6)	0.41
BP sys. (mmHg) median (Q1; Q3)			
(5)	98.9 (86.6; 110.2)	109.4 (105.8; 110.9)	0.10
(6)	96.0 (84.5; 102.7)	97.0 (88.4; 118.4)	0.48
(7)	106.1 (86.6; 119.9)	97.2 (81.7; 105.1)	0.62
(8)	92.2 (78.8; 111.7)	97.2 (81.7; 105.1)	0.75
(9)	90.9 (77.2; 116.4)	91.2 (88.1; 92.8)	0.51
BP diast. (mmHg) median (Q1; Q3)			
(5)	63.8 (56.6; 65.8)	67.4 (67.1; 68.5)	0.09
(6)	63.8 (57.7; 73.3)	67.2 (62.6; 70.3)	0.06
(7)	66.81 (52.8; 66.5)	70.1 (61.9; 76.2)	0.18
(8)	65.8 (59.8; 66.3)	61.4 (55.7; 64.5)	0.13
(9)	65.6 (61.7; 68.5)	60.5 (56.3; 62.0)	0.09
CVP / PAP (mmHg) median (Q1; Q3)			
(5)	16.2 (14.1; 21.6)	14.2 (9.4; 16.0)	0.04
(6)	14.3 (10.6; 19.2)	10.9 (9.7; 14.3)	0.03
(7)	17.11 (11.3; 19.1)	11.3 (8.7; 13.1)	0.03
(8)	11.9 (8.1; 18.2)	10.1 (8.5; 15.7)	0.15
(9)	11.5 (8.3; 13.1)	10.9 (8.5; 11.7)	0.70
ICU length of stay (d) median (Q1; Q3)	4.3 (3.4; 7.5)	3.4 (3.2; 5.1)	0.04
Maximal catecholamine index (µg/kg/min); median (Q1; Q3)	5.6 (4.6; 6.8)	3.4 (2.8; 4.1)	0.042
Respiratorysupport time (h); median (Q1; Q3)	13.9 (3.7; 18.5)	9.3 (7.0; 13.2)	0.03
Hospital stay; median (Q1; Q3)	17.2 (9.8; 26.5)	15.6 (12.1; 20.6)	0.44
Postoperative pleural effusions duration (days); median (Q1; Q3)	15.2 (14.2; 21.4)	12.5 (10.5; 17.6)	0.05

Data are presented as mean (SD) or median (Q1:Q3). Timepoint (5) after CPB discontinuation, (6) 4 h after surgery, (7) 8 h after surgery, (8) 12 h after surgery, (9) 24 h after surgery. *CPB* cardio-pulmonary by-pass, *NO* nitric oxide, *PCICU* pediatric cardiac intensive care unit

### Quantitative analysis of plasma factors

Based on the screening of proteome profilers, 19 analytes that were detectable at all time points were selected for high-sensitivity measurements: IL-10, IL-1b, TNF-a, GM-CSF, IL-6, IL-8, SDF-1, VEGF, IL-1ra, MMP-8, pentraxin-3, prolactin, NT-proBNP, CK-MB, troponin I, TIMP-4, angiopoietin-2, insulin, and leptin. Additionally, routine biochemical and hematologic parameters related to stress response, which differed in ANOVA and were accessible at all time-points (blood glucose concentration, lactates concentration, the number of neutrophils), were included in the further analysis.

**Fig. 2:**
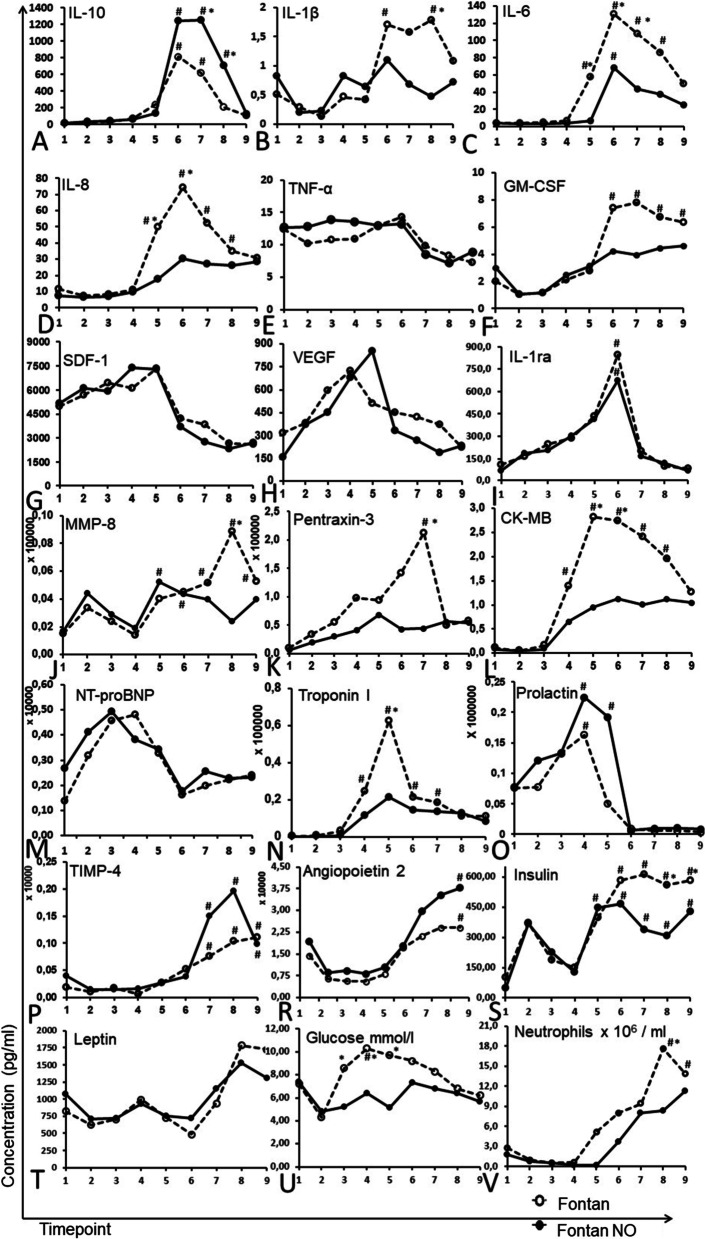
Quantitative Luminex-based analysis of plasma factors. Mean values of plasma factors concentrations. *Il-10* interleukin 10, *IL-1β* interleukin 1 beta, *Il-6* interleukin 6, *Il-8* interleukin 8, *TNFα* tumor necrosis factor α, *GM-CSF* Granulocyte–macrophage colony-stimulating factor, *SDF-1* stromal cell-derived factor 1, *VEGF* Vascular endothelial growth factor, *IL-1ra* interleukin-1 receptor antagonist, *MMP8* matrix metalloproteinase-8, *CK-MB* creatine kinase myocardial band, *NT-proBNP* N-terminal prohormone of brain natriuretic peptide, *TIMP-4* tissue inhibitor of metalloproteinase-4. Significant differences in post hoc testing were marked: *p < 0.05, between groups in corresponding timepoints; ^#^*p* < 0.05 between a reference (preoperative sample No 1) value and analyzed timepoint value

Fluctuations in measured cytokines and cardiac injury markers were observed in both the Fontan and Fontan-NO groups (Fig. 2., Additional file 2: Table S2). The Fontan-NO group had significantly higher levels of anti-inflammatory IL-10 after CBP. In contrast, the levels of pro-inflammatory IL-1 β, IL6, and IL-8 were significantly reduced compared to the Fontan group (Fig. 2). Additionally, patients in the Fontant-NO group had significantly lower levels of MMP-8, Pentraxin-3, CK-MB, and Troponin I.

A significant increase in insulin concentration was observed within the Fontan group compared to the preoperative level (Fig. 2S). In contrast, insulin concentration was lower in the Fontan-NO group and the difference was significant at time points 4 and 5 (Fig. 2S). A comparison between groups showed a significantly higher glucose concentration in the Fontan group at time points 3, 4 and 5 (Fig. 2U). As expected, the number of circulating neutrophils increased during surgery in both groups, although the rise was significant only within the Fontan group in ime-points 5 (*p* < 0.001) and 6 (*p* < 0.001) compared to the baseline level. In addition, a significant difference in neutrophil numbers was noted between groups (*p* = 0.011) 12 h after surgery. The remaining analytes (TNFα, NTproBNP, SDF 1, VEGF, leptin) showed insignificant variable characteristics and differences within and between groups (Fig. 2 and Additional file 2: Table S2).

Stepwise multiple regression analysis was performed to find factors related to early clinical outcomes, such as ICU time, maximal catecholamine index, respiratory support time, and duration of effusions. The results are presented in Table 2. There was a correlation between ICU time and respiratory support time and inflammatory factors (Il-6 and IL-8), while the catecholamine index and the time of effusion were related to the degree of myocardial damage (CK-MB, TnI).

**Table 2:** Results of stepwise multiple linear regression analysis predicting early clinical course

Dependent variable	Predictors	*β*	*P*
ICU time Adjusted *R*^2^ = 0,22	6 IL-6	0.52	0.012
CAI max Adjusted *R*^2^ = 0.67	5 CKMB	0.46	0.0008
	5 TnI	0.65	0.00002
Respiratory support time Adjusted *R*^2^ = 0.15	6 IL-8	0.43	0.03
Effusion’s duration Adjusted *R*^2^ = 0.18	5 TnI	0.41	0.04

All factors found to be significant in RMANOVA analysis were taken into the multiple linear regression models. *ICU time* intensive care unit hospitalization time, *CAI max* maximal catecholamine index, *6 IL-6* interleukin 6 concentration at ICU (4 h after surgery), 6 IL8 interleukin concentration at ICU (4 h after surgery), *5 CKMB* creatine kinase myocardial isoenzyme activity after by-pass discontinuation, *5 TnI* troponin I after by-pass discontinuation

## Discussion

To our knowledge, this study is the first to demonstrate the effects of inhaled NO on inflammatory and metabolic stress responses and myocardial and lung protection in patients during Fontan surgery. The influence of NO was clinically significant, as seen with the shortened respiratory support time, lower catecholamine index, and shorter time in intensive care. Furthermore, we showed NO's protective, anti-inflammatory, and metabolic stress-relieving effects through proteomic analysis of inflammatory cytokines, heart and lung damage indicators, and metabolic markers.

Early mortality after Fontan surgery is low and reported as 1–7% [22, 23]. However, Fontan-specific postoperative problems still contribute to prolonged postoperative recovery [24–26].Heart surgery with cardiopulmonary bypass (CPB) induces a tremendous stress reaction by activating many biological cascades [27]. This is mainly due to surgical trauma, blood contact with foreign materials, ischemia/reperfusion, abnormal shear stress, and hypothermia. Numerous strategies have been examined to alleviate or eliminate the undesirable effects of CPB. Apart from anesthetic methods, steroid administration and ultrafiltration are most popular in clinical practice [28, 29]. These methods aim to inhibit the synthesis of pro-inflammatory cytokines or eliminate them from the circulatory system. All patients enrolled in the present study received methylprednisolone.

As expected, compared to the reference point (preoperative sample 1), significant inflammatory stimulation, anti-inflammatory responses, and fluctuations of metabolic stress and tissue malperfusion indicators were observed in both groups. However, patients who received NO inhalation had significantly lower levels of pro-inflammatory cytokines (IL-8, IL-6, IL-1β) and higher levels of anti-inflammatory IL-10 (Fig. 2). The effect was clinically effective in Fontan-NO patients with significantly shorter intubation time and shorter time in the ICU (Table 1). Multiple regression analysis showed that the clinical outcomes correlated with IL-6 and Il-8 serum levels (Table 2).

Cecchia et al. demonstrated corresponding results. They observed lower morbidity and shorter respiratory support in children with tetralogy of Fallot receiving NO inhalation (14). IL-6 is key to the inflammatory response features; among others, it stimulates the interaction between neutrophils and cardiomyocytes, inducing myocardial damage after reperfusion [30]. IL-8 regulates trans-endothelial neutrophil migration and neutrophil-mediated tissue injury [31]. The concentrations of both IL-6 and IL-8 were significantly lower in patients receiving NO inhalation. Fontan-NO patients had higher levels of anti-inflammatory cytokine IL-10 (Fig. 2A), a crucial cytokine that inhibits pro-inflammatory cytokines and mediates protection of the microcirculation against the harmful activity of free radicals and proteases [32]. The heart-protective properties of exogenous IL-10 have been shown in surgical models and laboratory animals through improved ventricle function, reduced fibrotic remodelling, and lower mortality [33]. In our study, children who received NO had significantly reduced activity of enzymes which reflected cardiac injury (Troponin I and CK-MB) (Fig. 2L and N), suggesting that NO maintained the integrity and function of the myocardium during the critical reperfusion period.

Patients who received NO inhalation had a lower catecholamine index and propensity to shorter effusions. What is more, heart dysfunction indicators were coupled with CAI and effusions time in multiple regression models (Table 2.). In overloaded single ventricle conditions, an essential role of exogenous NO was shown in ischemia-induced preconditioning, cytoprotection, and resistance to ischemia–reperfusion injury of the heart [34, 35]. In our study, exogenous NO inhaled into the oxygenator flowed with oxygenated blood through the aortic cannula to the ascending aorta and directly to the coronary circulation. Although it is primarily inactivated in the blood by binding to oxy- and deoxyhemoglobin, a circulating pool of bioavailable NO was sufficient to improve and optimize perfusion [36].

Factors that reflect the general response to stress and tissue perfusion disturbances (prolactin, lactates, glucose, leptin, insulin, pentraxin-3, MMP-8, TIMP-4) were up-regulated in both groups (Fig. 2). A lower insulin level in the Fontan-NO group remains in line with a less pronounced inflammatory response, lower Il-6 and Il-8 levels, and higher Il-10 levels [37]. Although the population characteristics (age, gender, dominant ventricle anatomy, preoperative oxygenation, CPB time, and cross-clamp time) were comparable in both groups, lactate and glucose concentrations were significantly lower in patients receiving NO inhalation (Table 1). No difference was noted in heart rate and systolic and diastolic blood pressure. However, a central venous pressure reflecting pulmonary artery pressure after Fontan surgery was significantly lower within the first 8 h in Fontan-NO group. These results reflect a less pronounced stress response in Fontan-NO patients and better protection of pulmonary and coronary circulations. The results also suggest the rationale of maintaining NO inhalation following CPB and extubation (i.e. through nasal cannula).

MMP-8, a metalloproteinase related to neutrophil activity, was most elevated in both groups after reperfusion of the heart and lungs; however, its activity was significantly lower in the Fontan-NO group (Fig. 2). This corresponds with the reduced Il-6 and Il-8 levels and the lower number of mobilized neutrophils in the Fontan-NO group (30, 31). The counteracting MMP-8 tissue metalloproteinase inhibitor TIMP4 followed the dynamics of MMP-8 and was significantly higher in Fontan-NO patients. Ischemia–reperfusion injury of the lungs during CBP involves infiltrating the tissue by neutrophils and releasing cytokines and other harmful mediators like MMPs and TIMPs [38]. IL-6, Il-8, and TNFα increase the expression and release of MMPs [39]. Furthermore, MMP-8 has been shown to contribute to acute lung injury in the postoperative period [40]. The improved clinical response in the Fontan-NO patients implied better lung protection, which corresponds to the functional readouts.

A potential limitation of the study was that not all patients consented to blood sample collection.Although proteomic analysis methods were applied, the concentration of certain analytes was not detectable at all time-points or was too low for the multiplex method. Additionally, we only measured several clinical variables that were accessible for all patients.

## Conclusions

Administration of NO to the CPB oxygenator during Fontan surgery reduced ventilatory support time, improved hemodynamics early after the Fontan procedure and shortened ICU time. These clinical effects were correlated with proteome changes reflecting the reduced expression of pro-inflammatory cytokines and increased production of anti-inflammatory factors, reduced activity of metalloproteinases and increased activity of tissue metalloproteinases inhibitors, lowered myocardial and lung injury indicators and lowered expression of proteins reflecting metabolic stress.

## Supplementary Information

**Additional file1: Table S1.** Milliplex kits (Merck Millipore) used for the quantitative measurement of selected analytes in plasma samples collected from patients.

**Additional file 2: Table S2. **Concentrations of investigated factors in the peripheral blood (pg/ml).

## Abbreviations

SIMV: Synchronized intermittent mandatory ventilation
CK-MB: Creatine kinase myocardial band
GM-CSF: Granulocyte macrophage colony stimulating factor
CPB: Cardiopulmonary bypass
IL-1ra: Interleukin1 receptor antagonist
IL-1β: Interleukin 1 beta
IL-10: Interleukin 10
IL-6: Interleukin 6
IL-8: Interleukin 8
MMP8: Matrix metalloproteinase-8
NO: Nitric oxide
NT-proBNP: N-terminal prohormone of brain natriuretic peptide
SDF-1: Stromal cell-derived factor 1
TIMP-4: Tissue inhibitor of metalloproteinase 4
TNFα: Tumor necrosis factor α
VEGF: Vascular endothelial growth factor
