# Supplementary material for: The cardioprotective and anti-inflammatory effect of inhaled nitric oxide during Fontan surgery in patients with single ventricle congenital heart defects: a prospective randomized study
Source: J Intensive Care. 2022 Oct 13;10:48. doi: 10.1186/s40560-022-00639-y (PMC9558421; doi:10.1186/s40560-022-00639-y)
Supplement: Supplementary file 1 — Additional file1: Table S1. Milliplex kits (Merck Millipore) used for the quantitative measurement of selected analytes in plasma samples collected from patients. [file 40560_2022_639_MOESM1_ESM.docx]

## Additional file 1: Table S1. Milliplex kits (Merck Millipore) used for the quantitative measurement of selected analytes in plasma samples collected from patients.

| **Analyte** | **Milliplex kit** | **Cat. number** |
| --- | --- | --- |
| Angiopoietin-2 | Human Angiogenesis / Growth Factor Magnetic Bead Panel 1 | HAGP1MAG-12K |
| Insulin, leptin | Human Bone Magnetic Bead Panel | HBNMAG-51K |
| CKMB, Troponin I, NTpro-BNP | Human Cardiovascular Disease Panel 1 Magnetic Bead Kit | HCVD1MAG-67K |
| MMP-8 | Human Sepsis Panel 2 Magnetic Bead Panel | HSP2MAG-63K |
| SDF-1 | Human Cytokine/Chemokine Magnetic Bead Panel II | HCYP2MAG-62K |
| VEGF, IL-1ra | Human Cytokine/Chemokine Magnetic Bead Panel | HCYTOMAG-60K |
| Prolactin | Human Circulating Cancer Biomarker Magnetic Bead Panel 1 | HCCBP1MAG-58K |
| Pentraxin | Human Cardiovascular Disease (CVD) Magnetic Bead Panel 4 | HCVD4MAG-67K |
| HGF, TNFα, GM-CSF, IL-1β, IL-6, IL-8, IL-10 | High Sensitivity Human Cytokine Magnetic Bead | HSTCMAG-28SK |
| TIMP-4 | Human TIMP Magnetic Bead Panel 2 | HTMP2MAG-54K |
