# Supplementary material for: The cardioprotective and anti-inflammatory effect of inhaled nitric oxide during Fontan surgery in patients with single ventricle congenital heart defects: a prospective randomized study
Source: J Intensive Care. 2022 Oct 13;10:48. doi: 10.1186/s40560-022-00639-y (PMC9558421; doi:10.1186/s40560-022-00639-y)
Supplement: Supplementary file 2 — Additional file 2: Table S2. Concentrations of investigated factors in the peripheral blood (pg/ml). [file 40560_2022_639_MOESM2_ESM.docx]

## Additional file 2: Table S2. Concentrations of investigated factors in the peripheral blood (pg/ml).

|  | **Timepoint** | **Fontan** | | | | | **Fontan NO** | | | | |  |
| --- | --- | --- | --- | --- | --- | --- | --- | --- | --- | --- | --- | --- |
|  |  | **MEAN** |  | | **SD** | | **MEAN** | |  | **SD** | |  |
| **IL-10**  **F_T_=36.4; p<0.001**    **F_I_=5.16; p<0.0001** | **1** | 21.01 | ± | | 26.72 | | 31.93 | | ± | 17.65 | |  |
|  | **2** | 32.00 | ± | | 68.57 | | 22.78 | | ± | 9.12 | |  |
|  | **3** | 46.94 | ± | | 119.70 | | 15.42 | | ± | 16.60 | |  |
|  | **4** | 69.14 | ± | | 205.99 | | 18.05 | | ± | 18.72 | |  |
|  | **5** | 222.23 | ± | | 1511.21 | | 127.24 | | ± | 137.13 | |  |
|  | **6** | 1027.72 | ± | | 663.41 **#** | | 1139.88 | | ± | 843.79 **#** | |  |
|  | **7** | 539.79 | ± | | 494.81 **#** | | 1125.38 | | ± | 611.24 **#** * | |  |
|  | **8** | 259.57 | ± | | 103.14 | | 653.00 | | ± | 341.71 **# *** | |  |
|  | **9** | 117.19 | ± | | 79.27 | | 83.32 | | ± | 129.79 | |  |
| **IL-1b**    **F_T_=9.8; p<0.001**    **F_I_=4.2; p<0.001** | **1** | 0.51 | ± | | 0.45 | | 0.82 | | ± | 0.98 | |  |
|  | **2** | 0.29 | ± | | 0.27 | | 0.20 | | ± | 0.19 | |  |
|  | **3** | 0.14 | ± | | 0.08 | | 0.23 | | ± | 0.18 | |  |
|  | **4** | 0.47 | ± | | 0.44 | | 0.83 | | ± | 0.61 | |  |
|  | **5** | 0.42 | ± | | 0.36 | | 0.64 | | ± | 0.47 | |  |
|  | **6** | 1.71 | ± | | 1.24 **#** | | 1.10 | | ± | 0.86 | |  |
|  | **7** | 1.58 | ± | | 2.04 | | 0.68 | | ± | 0.64 | |  |
|  | **8** | 1.78 | ± | | 2.19**#** | | 0.47 | | ± | 0.43 ***** | |  |
|  | **9** | 1.08 | ± | | 1.33 | | 0.71 | | ± | 0.59 | |  |
| **IL-6**    **F_T_=32.4; p<0.001**    **F_I_=4.9; p<0.001** | **1** | 4.41 | ± | | 2.55 | | 3.88 | | ± | 4.96 | |  |
|  | **2** | 3.90 | ± | | 2.44 | | 3.20 | | ± | 3.05 | |  |
|  | **3** | 4.78 | ± | | 3.42 | | 3.34 | | ± | 3.30 | |  |
|  | **4** | 6.66 | ± | | 6.83 | | 3.91 | | ± | 4.50 | |  |
|  | **5** | 57.75 | ± | | 82.21 **#** | | 7.11 | | ± | 6.24 ***** | |  |
|  | **6** | 130.57 | ± | | 130.64 **#** | | 68.17 | | ± | 81.64 **#** * | |  |
|  | **7** | 108.33 | ± | | 112.66 **#** | | 43.76 | | ± | 33.64 ***** | |  |
|  | **8** | 86.14 | ± | | 89.55 **#** | | 37.62 | | ± | 40.59 | |  |
|  | **9** | 49.48 | ± | | 41.02 **#** | | 25.16 | | ± | 24.13 | |  |
| **IL-8**    **F_T_=24.8; p<0.001**    **F_I_=6.07; p<0.001** | **1** | 11.60 | ± | | 9.58 | | 6.54 | | ± | 4.62 | |  |
|  | **2** | 6.99 | ± | | 3.54 | | 5.27 | | ± | 1.72 | |  |
|  | **3** | 6.55 | ± | | 2.71 | | 4.63 | | ± | 1.92 | |  |
|  | **4** | 8.31 | ± | | 7.79 | | 8.05 | | ± | 0.26 | |  |
|  | **5** | 50.03 | ± | | 89.61 **#** | | 69.82 | | ± | 19.79 ***** | |  |
|  | **6** | 74.22 | ± | | 64.88 **#** | | 47.36 | | ± | 31.60 ***** | |  |
|  | **7** | 52.19 | ± | | 43.11 **#** | | 33.10 | | ± | 20.91 | |  |
|  | **8** | 35.05 | ± | | 26.24 **#** | | 22.10 | | ± | 12.61 | |  |
|  | **9** | 33.04 | ± | | 36.81 | | 25.28 | | ± | 13.72 | |  |
| **TNF-α**    **F_T_=4.9; p<0.01**    **F_I_=0.8; p<0.58** | **1** | 12.44 | ± | | 4.35 | | 12.69 | | ± | 8.76 | |  |
|  | **2** | 10.26 | ± | | 3.07 | | 19.30 | | ± | 18.65 | |  |
|  | **3** | 10.87 | ± | | 4.32 | | 18.34 | | ± | 27.10 | |  |
|  | **4** | 10.83 | ± | | 4.80 | | 18.04 | | ± | 34.99 | |  |
|  | **5** | 16.25 | ± | | 6.97 | | 26.40 | | ± | 116.61 | |  |
|  | **6** | 14.32 | ± | | 5.46 | | 13.21 | | ± | 4.63 | |  |
|  | **7** | 9.85 | ± | | 5.08 | | 8.49 | | ± | 4.03 | |  |
|  | **8** | 8.40 | ± | | 3.81 | | 7.20 | | ± | 2.87 | |  |
|  | **9** | 7.84 | ± | | 4.70 | | 9.60 | | ± | 4.42 | |  |
| **GM-CSF**    **F_T_= 16.4; p<0.001**    **F_I_= 3.38; p=0.16** | **1** | 1.98 | ± | | 0.94 | | 3.00 | | ± | 3.79 | |  |
|  | **2** | 1.03 | ± | | 0.79 | | 1.05 | | ± | 0.79 | |  |
|  | **3** | 1.19 | ± | | 1.12 | | 1.17 | | ± | 0.81 | |  |
|  | **4** | 2.12 | ± | | 3.51 | | 2.47 | | ± | 3.54 | |  |
|  | **5** | 2.78 | ± | | 3.39 | | 3.11 | | ± | 2.92 | |  |
|  | **6** | 7.38 | ± | | 9.86 **#** | | 4.23 | | ± | 6.92 | |  |
|  | **7** | 7.77 | ± | | 9.78 **#** | | 3.94 | | ± | 5.65 | |  |
|  | **8** | 6.71 | ± | | 8.71 **#** | | 4.43 | | ± | 5.66 | |  |
|  | **9** | 6.32 | ± | | 7.10 **#** | | 4.62 | | ± | 5.37 | |  |
| **SDF-1**    **F_T_= 6.0; p<0.01**    **F_I_= 0.58; p=0.51** | **1** | 5158.18 | ± | | 847.29 | | 4986.51 | | ± | 888.81 | |  |
|  | **2** | 6094.99 | ± | | 1586.50 | | 5708.22 | | ± | 1044.87 | |  |
|  | **3** | 5914.29 | ± | | 2215.09 | | 6424.47 | | ± | 851.75 | |  |
|  | **4** | 7388.67 | ± | | 2352.49 | | 6117.08 | | ± | 2045.19 | |  |
|  | **5** | 7309.33 | ± | | 3084.61 | | 7363.08 | | ± | 5223.91 | |  |
|  | **6** | 3696.24 | ± | | 1436.42 | | 4239.43 | | ± | 2161.18 | |  |
|  | **7** | 2739.84 | ± | | 827.94 | | 3843.93 | | ± | 1967.15 | |  |
|  | **8** | 2335.75 | ± | | 667.57 | | 2688.08 | | ± | 673.72 | |  |
|  | **9** | 2719.69 | ± | | 586.42 | | 2597.08 | | ± | 457.64 | |  |
| **VEGF**    **F_T_= 4.3; p<0.15**    **F_I_= 0.9; p=0.43** | **1** | 191.66 | ± | | 97.19 | | 142.41 | | ± | 117.08 | |  |
|  | **2** | 174.10 | ± | | 112.14 | | 116.69 | | ± | 31.13 | |  |
|  | **3** | 185.38 | ± | | 145.02 | | 92.97 | | ± | 56.01 | |  |
|  | **4** | 170.62 | ± | | 175.62 | | 107.40 | | ± | 115.13 | |  |
|  | **5** | 166.06 | ± | | 106.52 | | 185.93 | | ± | 230.71 | |  |
|  | **6** | 404.47 | ± | | 286.68 | | 282.85 | | ± | 304.47 | |  |
|  | **7** | 411.38 | ± | | 268.23 | | 183.87 | | ± | 128.29 | |  |
|  | **8** | 324.93 | ± | | 229.46 | | 133.54 | | ± | 107.30 | |  |
|  | **9** | 216.06 | ± | | 179.83 | | 230.09 | | ± | 300.80 | |  |
| **IL-1ra**  **F_T_= 5.0; p<0.001**    **F_I_= 0.09; p=0.9** | **1** | 111.42 | ± | | 136.63 | | 75.30 | | ± | 48.26 | |  |
|  | **2** | 73.18 | ± | | 78.51 | | 70.93 | | ± | 59.13 | |  |
|  | **3** | 73.30 | ± | | 68.93 | | 65.54 | | ± | 28.62 | |  |
|  | **4** | 53.34 | ± | | 39.26 | | 52.24 | | ± | 27.81 | |  |
|  | **5** | 170.95 | ± | | 332.57 | | 85.10 | | ± | 20.61 | |  |
|  | **6** | 1330.45 | ± | | 1422.82# | | 620.25 | | ± | 621.43# | |  |
|  | **7** | 248.41 | ± | | 334.93 | | 173.55 | | ± | 210.57 | |  |
|  | **8** | 101.59 | ± | | 62.73 | | 160.40 | | ± | 141.45 | |  |
|  | **9** | 89.24 | ± | | 58.14 | | 49.83 | | ± | 35.03 | |  |
| **MMP-8**    **F_T_= 49.6; p<0.015**    **F_I_= 0.35; p=0.02** | **1** | 1483.92 | ± | | 949.31 | | 1660.08 | | ± | 830.99 | |  |
|  | **2** | 3368.10 | ± | | 1875.43 | | 4401.64 | | ± | 1712.37 | |  |
|  | **3** | 2379.22 | ± | | 1450.73 | | 2862.28 | | ± | 1365.14 | |  |
|  | **4** | 1423.03 | ± | | 714.53 | | 1906.54 | | ± | 7308.05 | |  |
|  | **5** | 4022.12 | ± | | 1987.20 | | 5228.15 | | ± | 2551.44# | |  |
|  | **6** | 4498.40 | ± | | 1596.40# | | 4352.09 | | ± | 2073.22 | |  |
|  | **7** | 5158.50 | ± | | 1095.48# | | 3944.15 | | ± | 1943.36 | |  |
|  | **8** | 8882.59 | ± | | 1293.35# | | 2395.06 | | ± | 1146.18* | |  |
|  | **9** | 5263.82 | ± | | 727.34# | | 3974.22 | | ± | 967.23 | |  |
| **Pentraxin-3**    **F_T_= 2.9; p<0.003**    **F_I_= 1.8; p=0.04** | **1** | 10434.6 | ± | | 5293.39 | | 6183.2 | | ± | 3745.27 | |  |
|  | **2** | 34281.0 | ± | | 3783.72 | | 19231.2 | | ± | 4235.83 | |  |
|  | **3** | 54956.1 | ± | | 5924.72 | | 30019.4 | | ± | 4156.83 | |  |
|  | **4** | 97685.0 | ± | | 6792.65 | | 41198.8 | | ± | 4529.30 | |  |
|  | **5** | 94298.3 | ± | | 10828.10 | | 68272.6 | | ± | 10802.50 | |  |
|  | **6** | 141300.0 | ± | | 270742.00 | | 42904.3 | | ± | 58369.74 | |  |
|  | **7** | 212797.5 | ± | | 425379.67# | | 44404.8 | | ± | 18473.29* | |  |
|  | **8** | 49859.4 | ± | | 44410.66 | | 56444.0 | | ± | 103986.56 | |  |
|  | **9** | 58270.1 | ± | | 60460.28 | | 54221.4 | | ± | 70175.99 | |  |
| **CK-MB**        **F_T_= 17.5; p<0.001**    **F_I_= 3.8; p=0.001** | **1** | 10303.5 |  | | 4428.05 | | 8129.5 | |  | 6866.63 | |  |
|  | **2** | 4477.2 | ± | | 4136.18 | | 4294.7 | | ± | 3740.45 | |  |
|  | **3** | 15938.1 | ± | | 21557.65 | | 8397.6 | | ± | 10362.68 | |  |
|  | **4** | 138922.7 | ± | | 72503.96# | | 65525.8 | | ± | 69084.26 | |  |
|  | **5** | 282871.4 | ± | | 185800.77# | | 95412.3 | | ± | 64421.39* | |  |
|  | **6** | 274215.6 | ± | | 189794.75# | | 111962.3 | | ± | 80547.91* | |  |
|  | **7** | 240680.4 | ± | | 179529.06# | | 100520.4 | | ± | 44530.74 | |  |
|  | **8** | 196057.5 | ± | | 163556.86# | | 111480.8 | | ± | 36477.99 | |  |
|  | **9** | 126421.8 | ± | | 98983.96 | | 104233.5 | | ± | 25802.80 | |  |
| **NT-proBNP**      **F_T_= 2.5; p<0.01**    **F_I_= 0.22; p=0.98** | **1** | 1379.83 | ± | | 2240.08 | | 2679.50 | | ± | 1965.17 | |  |
|  | **2** | 3181.19 | ± | | 6662.70 | | 4130.71 | | ± | 5703.60 | |  |
|  | **3** | 4586.68 | ± | | 8826.73 | | 4944.67 | | ± | 6677.17 | |  |
|  | **4** | 4825.15 | ± | | 8439.77 | | 3828.19 | | ± | 4266.75 | |  |
|  | **5** | 3267.67 | ± | | 5926.12 | | 3425.77 | | ± | 3461.99 | |  |
|  | **6** | 1620.42 | ± | | 1467.80 | | 1758.38 | | ± | 1748.80 | |  |
|  | **7** | 1983.47 | ± | | 1712.59 | | 2550.63 | | ± | 1776.06 | |  |
|  | **8** | 2221.62 | ± | | 1256.43 | | 2284.54 | | ± | 1004.29 | |  |
|  | **9** | 2406.22 | ± | | 1371.19 | | 2301.52 | | ± | 661.39 | |  |
| **Troponin I**    **F_T_= 38.6; p<0.0001**    **F_I_= 10.1; p=0.001** | **1** | 170.82 | ± | | 114.58 | | 149.91 | | ± | 75.84 | |  |
|  | **2** | 501.27 | ± | | 590.22 | | 341.03 | | ± | 490.55 | |  |
|  | **3** | 2975.00 | ± | | 5289.56 | | 473.40 | | ± | 435.80 | |  |
|  | **4** | 24740.78 | ± | | 8409.16# | | 11614.08 | | ± | 9287.88 | |  |
|  | **5** | 62704.86 | ± | | 138284.89# | | 21309.43 | | ± | 24276.34* | |  |
|  | **6** | 21483.72 | ± | | 14985.03# | | 14383.18 | | ± | 7957.94 | |  |
|  | **7** | 18722.94 | ± | | 17385.01# | | 13808.45 | | ± | 9202.73 | |  |
|  | **8** | 11130.49 | ± | | 8410.66 | | 13008.37 | | ± | 6978.68 | |  |
|  | **9** | 11212.57 | ± | | 16552.87 | | 8384.43 | | ± | 4837.61 | |  |
| **Prolactin**    **F_T_= 38.5; p<0.001**    **F_I_= 4.6; p<0.0001** | **1** | 75863.73 | ± | | 30637.02 | | 77462.63 | | ± | 34059.15 | |  |
|  | **2** | 77308.02 | ± | | 22233.67 | | 120628.1 | | ± | 25412.96 | |  |
|  | **3** | 132520 | ± | | 15374.87 | | 133186.2 | | ± | 16529.61 | |  |
|  | **4** | 162682.8 | ± | | 14248.52# | | 224004 | | ± | 24900.55# | |  |
|  | **5** | 49978.14 | ± | | 33321.99 | | 192063.7 | | ± | 17127.42# | |  |
|  | **6** | 9271.684 | ± | | 1619.87 | | 6065.865 | | ± | 7182.04 | |  |
|  | **7** | 5768.911 | ± | | 14156.60 | | 9631.81 | | ± | 5225.80 | |  |
|  | **8** | 5774.517 | ± | | 12440.62 | | 10451.28 | | ± | 8861.97 | |  |
|  | **9** | 3255.98 | ± | | 18204.54 | | 8193.51 | | ± | 2072.23 | |  |
| **TIMP-4**    **F_T_= 3.4; p<0.001**    **F_I_= 1.24; p=0.007** | **1** | 188.26 | ± | | 96.86 | | 399.41 | | ± | 224.00 | |  |
|  | **2** | 104.13 | ± | | 88.20 | | 146.11 | | ± | 90.62 | |  |
|  | **3** | 172.33 | ± | | 91.87 | | 137.00 | | ± | 34.75 | |  |
|  | **4** | 57.73 | ± | | 21.68 | | 160.55 | | ± | 134.00 | |  |
|  | **5** | 269.15 | ± | | 131.61 | | 273.26 | | ± | 111.91 | |  |
|  | **6** | 515.79 | ± | | 306.46 | | 384.34 | | ± | 199.12 | |  |
|  | **7** | 755.71 | ± | | 258.31# | | 1499.06 | | ± | 746.94# | |  |
|  | **8** | 1031.92 | ± | | 896.61# | | 1964.34 | | ± | 808.17# | |  |
|  | **9** | 1109.45 | ± | | 347.89# | | 1840.24 | | ± | 860.60# | |  |
| **Angiopoietin**  **F_T_= 17.8; p<0.0001**    **F_I_= 0.95; p=0.47** | **1** | 14406.58 | ± | | 7478.59 | | 19310.45 | | ± | 11080.71 | |  |
|  | **2** | 6533.93 | ± | | 4318.49 | | 8521.16 | | ± | 4655.37 | |  |
|  | **3** | 5690.62 | ± | | 3662.39 | | 9242.43 | | ± | 4734.53 | |  |
|  | **4** | 5517.46 | ± | | 3235.16 | | 8138.37 | | ± | 4196.37 | |  |
|  | **5** | 8004.61 | ± | | 5991.96 | | 10348.26 | | ± | 5147.91 | |  |
|  | **6** | 17226.38 | ± | | 14611.13 | | 17805.61 | | ± | 14757.27 | |  |
|  | **7** | 21270.01 | ± | | 19055.86 | | 29883.21 | | ± | 23984.18 | |  |
|  | **8** | 24143.00 | ± | | 20555.63 | | 35356.30 | | ± | 32007.99 | |  |
|  | **9** | 24070.21 | ± | | 21383.83# | | 37863.85 | | ± | 37570.73# | |  |
| **Insulin**    **F_T_= 6.6; p<0.001**    **F_I_= 0.75; p=0.64** | **1** | 102.10 | ± | | 19.10 | | 48.05 | | ± | 44.11 | |  |
|  | **2** | 370.42 | ± | | 404.60 | | 367.51 | | ± | 17.09 | |  |
|  | **3** | 188.30 | ± | | 209.42 | | 255.86 | | ± | 10.56 | |  |
|  | **4** | 149.40 | ± | | 123.47 | | 127.43 | | ± | 12.97 | |  |
|  | **5** | 397.97 | ± | | 530.10 | | 446.03 | | ± | 66.06# | |  |
|  | **6** | 581.61 | ± | | 879.17# | | 467.93 | | ± | 363.78# | |  |
|  | **7** | 612.03 | ± | | 202.84# | | 336.96 | | ± | 253.13# | |  |
|  | **8** | 560.86 | ± | | 208.02# | | 309.46 | | ± | 229.66#* | |  |
|  | **9** | 580.24 | ± | | 647.76# | | 427.34 | | ± | 287.94#* | |  |
| **Leptin**    **F_T_= 1.8; p=0.18**    **F_I_= 0.44; p=0.94** | **1** | 824.85 | ± | | 902.13 | | 1078.07 | | ± | 384.87 | |  |
|  | **2** | 625.57 | ± | | 751.46 | | 705.52 | | ± | 260.61 | |  |
|  | **3** | 707.37 | ± | | 751.57 | | 717.71 | | ± | 276.05 | |  |
|  | **4** | 996.27 | ± | | 1035.43 | | 933.07 | | ± | 448.33 | |  |
|  | **5** | 729.41 | ± | | 758.06 | | 754.84 | | ± | 264.04 | |  |
|  | **6** | 485.67 | ± | | 366.91 | | 721.08 | | ± | 489.28 | |  |
|  | **7** | 936.48 | ± | | 717.90 | | 1154.53 | | ± | 768.88 | |  |
|  | **8** | 1781.94 | ± | | 2390.90 | | 1526.62 | | ± | 1208.01 | |  |
|  | **9** | 1725.80 | ± | | 1460.35 | | 1310.15 | | ± | 616.08 | |  |
|  | **1** | 7.42 | ± | | 4.24 | | 7.38 | | ± | 3.86 | |  |
| **Glucose** | **2** | 4.28 | ± | | 3.62 | | 4.78 | | ± | 2.76 | |  |
|  | **3** | 8.62 | ± | | 2.92 | | 5.22 | | ± | 2.68* | |  |
| **F_T_= 4.8; p<0.001** | **4** | 10.32 | ± | | 3.38# | | 6.48 | | ± | 3.18* | |  |
|  | **5** | 9.73 | ± | | 2.81 | | 5.13 | | ± | 2.83* | |  |
| **F_I_= 6.44; p<0.001** | **6** | 9.26 | ± | | 4.63 | | 7.34 | | ± | 3.8 | |  |
|  | **7** | 8.33 | ± | | 3.72 | | 6.83 | | ± | 4.2 | |  |
|  | **8** | 6.82 | ± | | 3.51 | | 6.46 | | ± | 3.2 | |  |
|  | **9** | 6.2 | ± | | 2.80 | | 5.75 | | ± | 2.4 | |  |
| **Neutrophils**  **FT= 20.9; p<0.001**  **FI= 8.8; p<0.007** | **1** | 2,81 | ± | 1.4 | | 1.81 | | ± | | | 0.6 | |
|  | **2** | 1.05 | ± | 1.3 | | 0,76 | | ± | | | 0,3 | |
|  | **3** | 0.57 | ± | 0.4 | | 0,52 | | ± | | | 0,2 | |
|  | **4** | 0.76 | ± | 0.6 | | 0,27 | | ± | | | 0,1 | |
|  | **5** | 5.15 | ± | 4.1 | | 0.23 | | ± | | | 0.1 | |
|  | **6** | 8.06 | ± | 4.2 | | 3.75 | | ± | | | 0.8 | |
|  | **7** | 9.4 | ± | 3.9 | | 7.99 | | ± | | | 1.7 | |
|  | **8** | 17.62 | ± | 6.1# | | 8.46 | | ± | | | 1.6* | |
|  | **9** | 13.7 | ± | 5.5# | | 11.35 | | ± | | | 0.7 | |

Presented values are mean ±SD. Results of post hoc testing: * p<0.05, between groups in corresponding timepoints; # p<0.05 between a reference (preoperative sample No 1) value and analyzed timepoint value. The values of F statistics in reference to time effect (FT) and interactive effect among time and group (FI) with corresponding p level values are given in the table for all measured parameters.
